# Evaluation of quality parameters of fried chicken chips containing different levels of roasted chickpea and corn flour

**DOI:** 10.1016/j.psj.2025.104905

**Published:** 2025-02-25

**Authors:** Orhan Ozunlu

**Affiliations:** Department of Food Engineering, Pamukkale University, Turkey, Denizli

**Keywords:** Chicken breast meat, Chips, Snack food, Drying, Frying

## Abstract

The effect of roasted chickpea and corn flour on the quality characteristics of chicken breast meat-based chips was evaluated in the present study. The chips are prepared in three formulations as follows: **CC-1** (5 % corn-15 % roasted chickpea flour), **CC-2** (10 % corn-10 % roasted chickpea flour), and **CC-3** (15 % corn-5 % roasted chickpea flour). The chips dough was placed into a single screw extruder, and the extrudates were dried for 1.5 h at 50 °C to get final moisture content of 10 %. Afterward, the extrudate samples were deep-fat fried for 30 s at 180 °C using a deep fryer, cooled on a baking tray for 20 min at room temperature, manually coated with ready chip spices as a flavoring in each baking tray and performed with chemical analyses such as proximate composition, pH value, TBARS (Thiobarbituric Acid Reactive Substances) value, carbonyl content and hardness value. The results showed that CC-1, and CC-2 samples had similar moisture, protein, and ash contents, while CC-3 samples were statistically higher in fat, and carbohydrate content compared to other samples (*P* < 0.05). The amounts of roasted chickpea and corn flour significantly affected the pH value of the chips (*P* < 0.05). TBARS values, and carbonyl contents showed statistically increments (*P* < 0.05) up to 60 days at room temperature were observed for all samples. A decrease in hardness was observed in all samples with storage, but an increase was observed with increasing corn flour level. The meat-based chips provided both an alternative to new gluten-free products for celiacs, and an important source of protein for athletes.

## Introduction

Adequate and balanced nutrition is a basic requirement for maintaining the growth, development, and wellness of individuals (Pereira and Vicente, 2013; Zhang et al., 2023). For a healthy and balanced diet, 55-60 % of daily calories intake should be provided from carbohydrates, the rest of the calories followed by 25-30 % from fats, and 10-15 % from proteins (Goswami et al., 2017; Lim, 2018). Although inadequate and imbalanced nutrition affects all age groups, they show especially more negative effects on infants, children and adolescents during their growth and development periods (Goswami et al., 2017). Most infant and child deaths have been caused by nutritional deficiencies caused by protein, energy, vitamin, and mineral deficiencies (Çamurdan, 2014).

Today, consumers cannot get enough animal-origin products due to economic issues, and they tried to supply this deficiency from cereals and cereal products in developing and undeveloped countries (Kowalska et al., 2020; Singh and Neelam, 2011). Although soy, peanut, pea, etc. products have high protein content, animal-based protein is superior to plant-based proteins in terms of biological value (Kumar et al., 2022; Marmonier et al., 2000; Nimitkeatkani et al., 2021). For a healthy life, an average of 50 g of protein approximately have been advised daily, and to fulfill this protein requirement, at least 40-% of this protein should be provided from animal-origin products according to nutritionists (Biesalski, 2005; Lorenzo et al., 2014; Pereira and Vicente, 2013). The consumption of chicken meat has increased throughout the world because it has high nutritional value (protein, vitamins, minerals low cholesterol, calorie, and fat contents), low price, is easy to prepare, flavor, and not any religious restrictions (Katiyo et al., 2020). Therefore, chicken meat has been primarily utilized as a raw material in the production of various products such as emulsified (sausage, sucuks), further processed (döners, nuggets, schnitzels, patty, etc.), and snack-type meat products (chips, crackers, biscuits, noodles, pretzel, sticks, etc.) in the poultry meat industry (Tan et al., 2018).

Recently, the dietary patterns of many societies have changed due to changing lifestyles, economic welfare, the urbanization process, busy schedules of working people, increasing number of young people (Berwal et al., 2013; İzci et al., 2011; Kastuhuri et al., 2017; Verma et al., 2014). Snack foods which are a part of ready-to-eat foods have started to become more popular especially among youth people as a result of the increasing product variety, the development of food technology, and the more attractive presentation of products via advertisements (Verma et al., 2023). Moreover, snack foods (dried nuts and fruits, chips, biscuits, sticks, breakfast cereal, and cereal products, etc.) could be defined as food products that are easy to carry, have a long shelf life, or are suitable for long storage and are highly crispy (Devalakshmi et al., 2010; Kastuhuri et al., 2017). Chips (especially potato and corn chips) are among the snack foods most preferred by consumers worldwide. Moreover, meat-based chips from beef, chicken or fish which can be alternative to potato and corn chips were found in global markets and scientific literature (Aykın-Dinçer et al., 2021; İzci et al., 2011; Kastuhuri et al., 2017, 2016; Özkaya, 2021).

Celiac disease is well-known autoimmune system disorder, caused by the digestion of gluten in the diet, showing significant effects on the gastrointestinal system (Lebwohl and Rubio-Tapia, 2021; Mulder et al., 2015). Moreover, celiac disease affected approximately 1-% of the world's population and has increased every year mainly in Australia, Europe, and North America (Raiteri et al., 2022). Celiacs should not consume such certain cereal products mainly wheat and barley have contained a high amount of gluten (Mulder et al., 2023). Accordingly, both scientists and the food industry have tried to produce a variety of gluten-free products (crackers, cookies, biscuits, pasta, cake, bread, etc.) for people suffering from celiac disease (Bender and Schönlechner, 2020; Nardo et al., 2019; Xu et al., 2020).

The objective of this study was to investigated the chicken breast meat potential for use in chip production and potential to be an alternative to gluten-free products as snack foods for celiac disease patients using three different rates of corn flour (5, 10, and 15 %) and roasted chickpea (5, 10, and 15 %) in chips.

## Materials and methods

### Materials

The chicken breast meat, corn flour, corn starch, salt, and sunflower oil were purchased from a supermarket (Migros Süpermarket) in Denizli, and the chicken breast was transported to Meat and Meat Technology Laboratory in Pamukkale University under the cold chain (at 4 °C). The roasted chickpea flour and Cajun seasoning mix (red pepper, basil, thyme, onion, garlic, and salt) were procured from a local dried nuts plant (Genç Dried Nuts, Serinhisar, Denizli, Türkiye) and a spices plant (Zengibar Spices Co., Ltd., Onikişubat, Kahramanmaraş, Türkiye), respectively. Moreover, Doypack coffee bags with aluminum (width/length: 8.5 cm x 14.5 cm; 3-layer packaging material [PET + aluminum + polythene]; product capacity: 250 g) as packaging materials were supplied from a manufacturing packaging plant (Eska Packaging Co., Ltd., Beylikdüzü, İstanbul, Türkiye) and all the reagents (analytical grade, purity ≥ 95 %) were acquired from Merck (Merck, Darmstadt, Germany).

### Preparation of chips dough, extrusion, and frying

The chicken breast meat was ground through a 3 mm plate using a meat grinder (Arnica Promeat Grande, Türkiye). 1.575 kg of chicken breast mince was divided into three different formulations and each 0.525 kg chicken mince in weight was placed separately in plastic caps. For the formulation of chips dough (0.7 kg chips dough, a total of 2.1 kg chips dough), all the ingredients then were mixed based on this formulation; 75 % (w/w) chicken breast mince, 1.5 % (w/w) salt, 3.5 % (w/w) corn starch. Afterward, roasted chickpea and corn flour were added to the dough mixture at three different ratios (5, 10, and 15 %), and three batches of chips dough were prepared: **(a)** CC-1 (5 % corn flour and 15 % roasted chickpea flour), **(b)** CC-2 (10 % corn flour and 10 % roasted chickpea flour), and **(c)** CC-3 (15 % corn flour and 5 % roasted chickpea flour). Prepared the dough were kneaded all the ingredients using a laboratory mixer (Karaca Mastermaid Chef Pro 1750 W, Karaca, Türkiye) for 5 min. Then, the extrusion was run under set conditions (20 min) up to desired temperature (50 °C) before the chips dough was placed into an single screw extruder (JY-MW Joyang Machinery, Shandong Joyang Machinery Co., Ltd., Chine; extruder dimensions: 25 × 2 × 2.5 m, total product capacity: 100-120 kg/h) fitted with a die nozzle of 4 mm diameter, and the extrudates were dried for 1.5 h at 50 °C to get final moisture content of 10 %. The extrudate samples (4.0 cm diameter, 0.1 cm thickness with average weight 3 g) were deep-fat fried for 30 s at 180 °C using a deep fryer (Tefal Filtra Pro Premium 4 L Digital FR5160, Tefal, France; **frying oil capacity:** 4 L), and dried with a paper towel to remove any excess fat. After cooling and seasonings, they were transferred to airtight containers for storage. And then, the chip samples were manually coated with ready chip spices as a flavoring in each baking tray.

### Air packaging of the chips-based chicken meat samples

After the fried chips samples were cooled under aseptic conditions, they (approximately, 20 g) were placed into packaging materials, and they were packed under air atmosphere using a sealing packaging machine (Koodmax Wozlo Psf-300 Sealing Packaging machine, Koodmax, Türkiye), and then the physicochemical (pH, TBARS value, carbonyl content, texture profile) of fried chicken chips (180 °C, 30 s) were tried to determine the oxidative stability of the chicken chips at the 0th, 15th, 30th, 45th and 60th days of storage intervals.

## Analysis

### Proximate composition

The proximate composition (moisture, protein, and ash) of chips-based chicken breast was analyzed as described by the AOAC (2006), while fat content in the samples was performed with solvent extraction using methanol and chloroform chemical solutions (1:2, V/V) at room temperature for 24 h according to Flynn and Brambert (1975) procedure. The total carbohydrate content in the chips samples was calculated by subtracting the moisture, protein, ash, and fat contents from 100.

### pH value

For pH measurement, 10 g of the chips samples were homogenized with 90 ml of distilled water using a homogenizer (HG-15A, WiseTis, Daihan Scientific Co. Ltd., Wonju, Korea) for 2 min. Afterward, the pH value of the chips samples was measured using a digital pH meter (Crison Basic 20, Spain). Before pH measurement, the pH probe was calibrated with buffers solutions of pH 4, 7 and 10 (Merck, Germany), respectively, at room temperature (Ergezer et al., 2018).

### TBARS value

To determine the oxidation of lipids in chips samples, TBARS (Thiobarbituric Acid Reactive Substances) analysis was conducted according to Ergezer and Serdaroğlu (2018) with slight modifications. Chip samples (5 g) were homogenized in 50 ml TCA (20 %, w/v) using a homogenizer (HG-15A, WiseTis, Daihan Scientific Co. Ltd., Wonju, Korea). The homogenate was filtered through a filter paper (No.1, Whatman International Ltd., Maidstone, England) and 5 ml of filtrate was taken into a screw- capped polypropylene tube. After adding 50 ml of TBA (0.02 M), the tubes were left in a boiling water bath (NB-5, Nüve, Ankara, Turkey) until a pink color appeared. The tubes were then cooled down to room temperature and used for absorbance measurement at 532 nm in a spectrophotometer (EMC- 11- UV, EMC LAB Instruments GmbH, Duisburg, Germany) using 1,1,3,3-tetra ethoxy propane as an external standard. TBARS values were calculated as mg of malonaldehyde per kg chips samples.

### Carbonyl content

The carbonyl contents were determined by using DNPH (dinitrophenylhydrazine) method described by Ferreira et al. (2017). Samples (1 g) were homogenized in 10 ml SPBS (20 mmol L − 1), containing 6 mol L − 1 NaCl (pH: 6.5) using a homogenizer (HG-15A, WiseTis, Daihan Scientific Co. Ltd., Wonju, Korea). Two portions of homogenates (0.2 ml) were taken and separately placed into Eppendorf tubes. After precipitating proteins with 1 ml of cold TCA (10 %), the tubes were centrifuged (Eppendorf MiniSpin, Hamburg, Germany) at 4200 × g for 5 min. One of the pellets was treated with 1 ml of HCl (2 mol L − 1) to determine protein concentration and the other pellet was treated with 1 ml of DNPH (0.2 %, w/v) in 2 mol L − 1 HCl to determine carbonyl concentration. After incubating at room temperature for 1 h, samples were precipitated with 1 ml of cold TCA and washed three times with 1 ml of a mixture of ethanol and ethyl acetate (1:1, v/v) to remove excess DNPH. The pellets were dissolved in 1.5 ml of SPBS containing 6 mol L − 1 guanidine HCl (pH: 6.5) and then centrifuged at 4200 × g for 2 min to remove insoluble fragments. The absorbance measurement was performed at 280 nm using bovine serum albumin as a standard and protein concentration was calculated. Carbonyl concentration was measured based on an absorption coefficient of 21.0 mM^−1^ cm^−1^ at 370 nm for protein hydra-zones. Results were expressed as nmol of carbonyl per mg of protein.

### Hardness parameters

Hardness analysis was performed to break the chip-based chicken breast samples using the Brookfield CT3 texture analyzer (Brookfield Engineering Laboratories Inc., CT3‐4500, Massachusetts, USA) found in the Unit Operation Laboratory in Department of the Food Engineering, Pamukkale University after a 15-day interval on 0, 15, 30, 45, and 60 days of storage at room temperature, and three-point test (TA-TPB) was used to determine the hardness properties of the chip samples. A total of ten measurements per replication were taken, and the average values were recorded. The pre-test, test, and post-test speed were adjusted to 2.50 mm/s, 1.50 mm/s, and 1.50 mm/s, respectively. Trigger load was 100 g.

### Statistical analysis

The effects of three different flour levels (5, 10, and 15 %) using corn and roasted chickpea flour on the physicochemical (proximate composition, pH, TBARS value, carbonyl content, and texture profile) of the chips-based chicken breast meat were performed statistical analysis containing one-way and two-way analysis of variance (ANOVA) as well as Duncan's Multiple Range Test by using SPSS for Windows (SPSS version 15.0 for Windows). A total of six-hundred chip samples (two-hundred chip samples for each group) and thirty Doypack coffee bags with aluminum (2 parallel x 3 batches x 5 storage days, ***n*** **=** 30) used for chicken breast meat-based chip production. The results were assessed with a significant level of *P* < 0.05 and there were presented as the mean ± standard error.

## Results and discussion

### Proximate composition

The proximate composition results of the chip samples, including moisture, protein, total fat, ash, and carbohydrate, are shown as a percentage in Table 1. The moisture content of the samples varied between 8.68 and 9.75 %. Although the moisture content was significantly (*P* < 0.05) higher for CC-1, there were no significant differences (*P* > 0.05) between CC-1 (9.75 %) and CC-2 (9.46 %). Also, a significant (*P* < 0.05) difference was observed between CC-2 (9.46 %) and CC-3 (8.68 %) samples. These differences in moisture content were probably owing to the different levels of corn and roasted chickpea flour used in the production of chip samples. The samples decreased (from 9.75 % to 8.68 %) as the level of corn flour increased in chip-based chicken samples, the moisture content decrease might be ascribed to the roasted chickpea flour having higher water holding capacity than the corn flour (Kaur and Singh, 2005; Nasiri et al., 2012; Yu et al., 2016). Increases in moisture content of redfish (a by-product of fillet processing from grass carp) meat-based fried snacks by the addition of different levels of red fish meat (0, 10, 20, and 30 %) were reported by Nawaz et al. (2019a). In another study, the addition of 1.79 % gelatin used in the formulation of the chicken meat-based snack, as mentioned by Bulut and Candoğan (2022), did not significantly change the moisture content compared to the control samples (*P* > 0.05).

**Table 1:** The proximate composition of chip samples-based chicken breast meat

Groups	Proximate composition				
	Moisture (%)	Protein (%)	Fat (%)	Ash (%)	Carbohydrate (%)
**CC-1**	9.75±0.24^a^	19.85±0.45^a^	6.67±0.13^c^	2.20±0.11^a^	61.53±0.26^c^
**CC-2**	9.46±0.21^a^	19.10±0.41^a^	7.13±0.24^b^	2.13±0.16^a^	62.18±0.24^b^
**CC-3**	8.68±0.17^b^	18.35±0.32^b^	7.74±0.25^a^	2.15±0.19^a^	63.08±0.23^a^

^a, b, c^: Mean values within the same column bearing different superscripts differ significantly (P<0.05).

**CC-1:** 75% chicken breast mince, 15% roasted chickpea flour, 5% corn flour, 3.5% corn starch, 1.5% salt; **CC-2:** 75% chicken breast mince, 10% roasted chickpea flour, 10% corn flour, 3.5% corn starch, 1.5% salt; **CC-3:** 75% chicken breast mince, 5% roasted chickpea flour, 15% corn flour, 3.5% corn starch, 1.5% salt

The protein content results obtained from the study are presented in Table 1. The protein content was significantly higher for CC-1 (19.85 %) samples followed by the CC-2 (19.10 %) and CC-3 (18.35 %) samples, respectively. The different corn and chickpea flour levels used in this study could affect the protein content; however, they were no statistically observed differences between CC-1 and CC-2 samples (*P* > 0.05). The results revealed that the protein content of the samples increased from 18.35 % to 19.85 %, as the increasing levels of chickpea flour in the chip samples. This increment considered that could be caused by the presence of high levels of protein in roasted chickpea flour. Nawaz et al. (2019b) reported that the addition of fish meat increased the protein content in snacks based on different levels (0, 10, 30, and 50 %) of wheat flour with the different amounts (0, 15, and 30 g; as per 100 g) of fish meat addition. Philomina (2022) observed moisture (2-4 %), fat (10-15 %), protein (18.91-32.81 %), and ash (2-7 %) content in snacks (chin-chin) produced from blends of the different flours (chicken and wheat flour).

The fat content of the chip samples is displayed in Table 1. The interaction between three different flour levels (5, 10, and 15 %) significantly affects the fat content of the fried (180 °C) chip samples (*P* < 0.05). The fat content was lower in the chip samples-based chicken breast meat with 15 % roasted chickpea and 5 % corn flour addition. The fat content was increased from 6.67 to 7.74 as a percentage (%) with the increasing incorporation of corn flour into chip samples, indicating the proximate composition difference between the corn and roasted chickpea flour. Because corn flour has low water retention, water evaporation creates cavities, which fill with oil during the frying process. Consequently, there was an opposite relationship between moisture and fat content; that is, the corn flour possibly absorbed more fat as the increasing levels of the corn flour increased during the frying process (Chen et al., 2019; Ngadi et al., 2007; Nasiri et al., 2012). Similar to this study, Nawaz et al. (2021) stated that the fat content increased (from 1.45 to 3.54 %) with the addition of the four different levels (0, 5, 10, and 15 %) of whole fish powder in baked snack products. In another study, the findings were observed by Manikandan et al. (2020), where fat content recorded ranged from 24.04 to 25.65 % in extruded snacks with different levels (0, 5, 10, and 15 %) of the shrimp shell powder however, there did not find significant differences among the samples (*P* > 0.05).

As shown in Table 1, the proximate composition for chip samples-based chicken breast meat with corn and roasted chickpea flour showed no significant difference in terms of ash content. However, the ash content was relatively found higher in the CC-1 samples (2.20 %), and this may be owing to the higher ash content in the roasted chickpea flour used in the formulation of the chip samples-based chicken breast meat. Baskar et al. (2022) determined that significantly increased the ash content for the four different levels (0, 2.5, 5, and 7.5 %) of the fish powder and the four different levels (0.25, 0.50, 0.75, and 1 %) shrimp head exudate in an extruded product until the storage of 90-days (*P* < 0.05). In another study, Sanusi et al. (2022), evaluated to influence on cooking treatments (untreated, boiling, and steaming), ingredient-mix three different infusion temperatures (30, 40, and 50 °C), and three different infusion duration (5, 10, and 15 min) in the chicken kilishi (ingredient-mix based dried chicken product produced from chicken breast meat) in terms of the ash content, and the ash content increased with infusion temperature and infusion time.

The carbohydrate content of the chip samples which prepared according to three different recipes were shown in Table 1. Concerning carbohydrate content, the means of all samples ranged from the lowest of 61.53 % (CC-1) to the highest of 63.08 % (CC-3). As seen in Table 1, although the carbohydrate content significantly increased with increasing amounts of added corn flour in the chip samples (*P* < 0.05), the protein and ash content increased as increasing levels of roasted chickpea flour in the chip samples due to proximate composition between corn and roasted chickpea flour.

Chudasama et al. (2019) produced functional fish (using eyes of Priacanthus hamrur which is commonly found on the South-East coast of India) crackers with three different flours (50 % rice flour, 50 % sago flour, and 50 % tapioca flour), and the carbohydrate content of samples found as 59.97, 48.05, and 57.77 %, respectively. In another study, the carbohydrate content of crispy corn with chicken feet flour (0, 15, 30, 45, and 60 %) gradually decreased with the increasing ratio of chicken feet flour (Hadi et al., 2022).

### pH value

The evaluation of the pH value during the storage stage (60 days, at room temperature) of the packaged chip samples-based chicken breast under air oxygen is shown in Table 2. The pH value of the chip samples-based chicken breast meat was significantly affected by storage time (*P* < 0.05), and they were decreased until day 30 of storage. As storage time increased from day 15 to day 30, there were no significant differences found in the CC-2, and CC-3 samples (*P* > 0.05). At whole storage periods (except on days 0 and 15), the pH value of the CC-1 and CC-3 samples showed a similar pattern, and the pH value of all samples significantly increased from day 30 to day 60 (*P* < 0.05). The pH value of the CC-3 samples was higher than other chip samples during the storage periods however, there were no significant differences found among the samples on day 60 of storage (*P* > 0.05). The pH value of all samples was decreased until day 30 of storage depending on a greater increase in the various peptides, and organic acids due to proteins in the meat being decomposed into some metabolites by the microorganisms (Özünlü, 2023), there were significantly increased from day 30 to day 60 (*P* < 0.05) because of microbiological changes (Dudnyk et al., 2018).

**Table 2:** The physicochemical properties (pH, TBARS value and carbonyl content) of chip samples-based chicken breast meat throughout the storage

pH value					
Groups	Storage times (days)				
	0	15	30	45	60
**CC-1**	5.87±0.01^bC^	5.84±0.02^abC^	5.83±0.01^aC^	5.90±0.01^aB^	5.95±0.02^aA^
**CC-2**	5.85±0.02^bB^	5.82±0.01^bC^	5.80±0.01^bC^	5.87±0.02^bB^	5.97±0.02^aA^
**CC-3**	5,91±0.01^aB^	5.87±0.01^aC^	5.85±0.02^aC^	5.93±0.01^aB^	6.01±0.03^aA^
**TBARS value (mg malondialdehyde/kg product)**					
**CC-1**	0.13±0.03^aC^	0.19±0.02^aB^	0.29±0.09^aA^	0.44±0.08^aA^	0.53±0.03^bA^
**CC-2**	0.15±0.05^aC^	0.22±0.06^aBC^	0.33±0.11^aB^	0.45±0.06^aB^	0.59±0.01^aA^
**CC-3**	0.18±0.10^aD^	0.26±0.04^aD^	0.38±0.07^aC^	0.51±0.03^aB^	0.64±0.04^aA^
**Carbonyl content (nmol carbonyl/mg protein)**					
**CC-1**	0.31±0.04^aE^	0.39±0.03^bD^	0.55±0.06^aC^	0.78±0.03^bB^	0.87±0.04^bA^
**CC-2**	0.35±0.02^aE^	0.43±0.05^aD^	0.59±0.07^aC^	0.86±0.02^aB^	1.01±0.05^aA^
**CC-3**	0.36±0.01^aE^	0.48±0.02^aD^	0.63±0.05^aC^	0.91±0.08^aB^	1.05±0.03^aA^

^a, b^: Mean values within the same column bearing different superscripts differ significantly (P<0.05).

^A, B, C, D, E^: Mean values within the same row bearing different superscripts differ significantly (P<0.05).

**CC-1:** 75% chicken breast mince, 15% roasted chickpea flour, 5% corn flour, 3.5% corn starch, 1.5% salt; **CC-2:** 75% chicken breast mince, 10% roasted chickpea flour, 10% corn flour, 3.5% corn starch, 1.5% salt; **CC-3:** 75% chicken breast mince, 5% roasted chickpea flour, 15% corn flour, 3.5% corn starch, 1.5% salt

Similarly, Çakmak et al. (2016) produced the baguette bread snacks containing chicken meat powder (10, and 15 %). The pH values of chicken meat powder (10 and 15 %)-based baguette bread significantly increased until the day 30th, then decreased up to the 75th day of the storage (*P* < 0.05). In another study, the pH value of battered and breaded snack products from large-sized Catla (Catla catla) fish meat was slightly increased from 6.50 to 6.79 during 18 days of refrigerated storage; however, this increment did not find (*P* > 0.05) significantly (Pawar et al., 2020).

### TBARS value

The effect of the three different flour levels (5, 10, and 15 %) on the TBARS value of the chip samples-based chicken breast meat was shown in Table 2. The initial values of the TBARS in CC-1, CC-2, and CC-3 samples were found to be 0.13±0.03, 0.15±0.05, and 0.18±0.10 mg malondialdehyde/kg product, respectively. As can be seen in Table 2, the TBARS value of the CC-1 samples was lower than those of CC-2, and CC-3 samples, and they had significantly similar TBARS values (*P* > 0.05) on each of the storage periods (except the 60th day). At the end of the storage, the CC-3 samples (0.64 mg malondialdehyde/kg product) had the highest TBARS value. Increased TBARS value has been related to the increasing formation of secondary products of lipid oxidation (mainly malondialdehyde etc.) with the progress of storage periods (up to days 60) at room temperature (Berardo et al., 2016). As the days passed, the TBARS value gradually increased until the end of the storage period however, the TBARS values in the chip samples have remained below the threshold value (<1 mg malondialdehyde/kg product) accepted by the various researchers (Özcan et al., 2018; Özünlü and Ergezer, 2022; Reitznerova et al., 2017) since the packaging materials had low water vapor and gas permeability.

These values are in agreement with those of Egbedi and Osibona (2021), who reported the TBARS value of 1.78-4.12 mg malonaldehyde/kg product ready-to-eat snack foods-based fish meat (*Tilapia guineensis*) during a three-week storage period. Kumar et al. (2016) stated that the threshold level of the TBARS value for chicken meat is 2 mg malondialdehyde per kg product; however, after 180-day storage at room temperature, the TBARS value of the chicken meat biscuits incorporated with wheat and oat bran remained lower than accepted the threshold value. Raja et al. (2014) also observed significant differences in the TBARS values of the aerobically packaged fish curls incorporated with different flours (corn, black gram, peanut flour), though the TBARS values were found lower than the threshold value (<1 mg malondialdehyde/kg product) suggested by (Berardo et al., 2016; Cakmak et al., 2016; Özünlü and Ergezer, 2022; Pawar et al., 2020) until 21 days of the storage, however, these values have remained above the threshold value (>1 mg malondialdehyde/kg product) at the end of the storage.

### Carbonyl content

The carbonyl content is a common indicator of protein oxidation, and the trend of change in carbonyl content is presented in Table 2. No significant difference was observed among the samples on days 0, and 30 (*P* > 0.05). Noteworthily, although the carbonyl content of the CC-3 samples was higher than those of the CC-1 and CC-2 samples (*P* < 0.05) regardless of roasted chickpea and corn flour levels, there were no significant differences found between CC-2 and CC-3 samples at each of the storage days (*P* > 0.05). As shown in Table 3, the carbonyl content of the CC-1, CC-2, and CC-3 samples were 0.31, 0.35, and 0.36 nmol carbonyl/mg protein, respectively, on day 0, which increased to 0.87, 1.01, and 1.05 nmol carbonyl/mg protein, respectively, on day 60. Accordingly, the increased rates of the carbonyl content in the chip samples were CC-3 (191.67 %) > CC-2 (188.57 %) > CC-1 (180.65 %) during the 60 days of storage. The increase in carbonyl content of the chip samples could be explained by the oxidation of some amino acids such as cysteine, methionine, arginine, and lysine which are more susceptible for producing carbonyl compounds during storage (Al-Dalali et al., 2022).

**Table 3:** Hardness results of chip samples-based chicken breast meat throughout the storage

Hardness value (g)					
Treatments	Storage times (days)				
	0	15	30	45	60
**CC-1**	3,230.11±24.75^bA^	3,222.63±21.12^bA^	3,207.08±35.47^bA^	3,205.48±20.19^bA^	3,076.57±17.85^cB^
**CC-2**	3,268.25±31.12^abA^	3,254.18±14.23^bA^	3,229.34±30.18^bAB^	3,219.96±23.31^abB^	3,163.24±25.49^bC^
**CC-3**	3,327.39±29,33^aA^	3,313.05±32.16^aA^	3,306.52±27.65^aA^	3,278.55±29.78^aAB^	3,214.71±33.25^aB^

^a, b, c^: Mean values within the same column bearing different superscripts differ significantly (P<0.05).

^A, B, C^: Mean values within the same row bearing different superscripts differ significantly (P<0.05).

**CC-1:** 75% chicken breast mince, 15% roasted chickpea flour, 5% corn flour, 3.5% corn starch, 1.5% salt; **CC-2:** 75% chicken breast mince, 10% roasted chickpea flour, 10% corn flour, 3.5% corn starch, 1.5% salt; **CC-3:** 75% chicken breast mince, 5% roasted chickpea flour, 15% corn flour, 3.5% corn starch, 1.5% salt

Monteiro et al. (2016) produced pasta fortified with different tilapia (*Oreochromis niloticus*) flour levels (0, 6, 12, 17, and 23 %), the carbonyl content of samples significantly increased during 21 days at 25°C, and tilapia flour levels affected the carbonyl content on each storage periods. In another study, the carbonyl content of the ostrich, beef, and chicken jerky snacks significantly (*P* < 0.05) increased from 5 to 15; from 7 to 20; from 3 to 5 nmol carbonyl per kg protein, respectively, during storage (room temperature, 9 months) (Zdanowska-Sasiadek et al., 2022).

### Hardness parameters

Table 3 shows the effect of the corn, and roasted chickpea flour on the hardness properties of the chip-based chicken breast meat samples during room temperature storage which portrays a significant effect (*P* < 0.05) of the storage time, and corn, and roasted chickpea flour treatments. Moreover, the hardness did not statistically change for CC-1 and CC-3 samples until 60 days of storage (*P* > 0.05). After 45th days of storage, the hardness had a significant difference among the samples (*P* < 0.05). On day 60, the addition of the corn flour to the chip samples formulation led to a significant change in the product (*P* < 0.05), making the sample crumbly. A decrease in the hardness was observed in the samples with storage however, there was an increase observed in the samples with an increase in the corn flour levels. This result may be attributed to the weak water and fat binding or adhesion capacities of the roasted chickpea flour used in chip dough (Kayacier and Singh, 2003; Pineda, 2007; Rababah et al., 2012). Bakare et al. (2020) analyzed the effect of edible fish meat, breadfruit, and wheat flour at different concentrations (from 0 to 40 % for edible fish meal and wheat flour; from 0 to 60 % for breadfruit) on the hardness characteristics of biscuits-based fish meat, and the hardness value of the samples significantly ranged from 48.23 to 2118.40 N (*P* < 0.05). A similar result was reported by Cakmak et al. (2016) in a study with different levels (10 and 15 %) of chicken meat and chicken meat powder in baguette bread as snacks foods, as chicken meat and chicken powder increased, they observed a significant increase in hardness value, but on the other hand, the hardness value of the samples indicated some fluctuate during storage (25 °C, 90 days). Furthermore, although the increase in the additional levels of mechanically deboned poultry meat to 30 % decreased the values of hardness value of snacks foods containing 20 % brewer's spent grain, there were no significant differences (*P* > 0.05) among the samples (Delic et al., 2020).

## Conclusion

Due to changing of eating habits among sociocultural changes in humans, there was an increase in the consumption of the various snack foods described as ready-to-eat functional food products. Formerly, potato and corn chips as snack foods are very popular in many countries, and they are commonly consumed by children and young people around the world. These products have very low protein, vitamin and mineral content and contrary are high in calorie and total fat and salt content. In this sense, both scientists and the meat industry are trying to develop together plant and/or animal-based protein sources based-snacks foods to get healthier products.

Within this context, the chicken breast could be used in the chip formulation and produced as a new snack food product for celiac disease, and some physicochemical of the chip samples were investigated during two months of storage at room temperature. These findings demonstrate a higher similarity in the moisture and protein content of the CC-1 and CC-2 samples than the CC-3 samples. The fat and carbohydrate content in the CC-1 samples was significantly lower than in the CC-2 and CC-3 samples (*P* < 0.05). The ash content was not notably influenced by the amounts of roasted chickpea (5, 10, and 15 %) and corn (5, 10, and 15 %) flour used to produce chip samples (*P* > 0.05). The pH value of the chip samples decreased until the 30th day of storage times, followed by subsequent increased with increasing storage times. Despite observing variations in TBARS values over storage times, the chip samples have consistently maintained their quality within acceptable threshold value (<1 mg malondialdehyde/kg product), highlighting the efficacy of the 3-layer packaging material employed. The carbonyl contents increased throughout the storage times, regardless of the roasted chickpea and corn flour levels added. As increasing levels of corn flour in the samples, they have resulted in improved textural properties. In conclusion, the developed chips samples increased the nutritional value (higher protein, vitamin, and mineral content), and they considered that could be alternatives to other snack foods such as potato and corn chips, crackers, cookies, pretzels, biscuits, etc. for the food industry. Moreover, this product thought that could be an alternative for individuals such as athletes who need concentrated protein.

## Disclosures

The author declares that there is no conflict of interest.
